# Comparison of the Qualitative and the Quantitative Risk Assessment of Hazardous Substances Requiring Management under the Occupational Safety and Health Act in South Korea

**DOI:** 10.3390/ijerph18031354

**Published:** 2021-02-02

**Authors:** Hyung-Il Moon, Sang-Woo Han, Saemi Shin, Sang-Hoon Byeon

**Affiliations:** Department of Health and Safety Convergence Science, Graduate School, Korea University, 145 Anam-ro, Seongbuk-gu, Seoul 02841, Korea; himoon86@hanmail.net (H.-I.M.); kompany11@daum.net (S.-W.H.); saemishin@naver.com (S.S.)

**Keywords:** qualitative risk assessment, quantitative risk assessment, control banding, hazardous substances requiring management, occupational safety and health act

## Abstract

The risk assessment of hazardous substances has become increasingly important for the efficient prevention and management of various diseases or accidents caused by increased amounts of hazardous substances in the workplace. In this study, risk assessment was conducted for 36 kinds of hazardous substances requiring management by using qualitative and quantitative risk assessments. Qualitative risk assessment was performed by multiplying the exposure level class by the hazard class according to the Korea Occupational Safety and Health Agency’s (KOSHA) Chemical Hazard Risk Management (CHARM). The quantitative risk assessment was followed by a four-step risk assessment system presented in the Guidelines for Hazard Risk Assessment of Chemicals (KOSHA GUIDE W-6-2016). In the quantitative assessments, we presented a new method of classifying risk levels into four steps, much like qualitative assessments. In this study, the quantitative risk assessment was considered difficult to predict through qualitative risk assessment. Therefore, it is necessary to perform a quantitative risk assessment after a qualitative risk assessment for a higher level of risk assessment.

## 1. Introduction

Harmful chemicals used in industrial sites exist in various forms from raw materials to by-products that are produced in the process and the use of these chemicals has steadily increased since the Industrial Revolution [1]. Each year, about 400 new substances are introduced in domestic industrial sites, but most of the direct victims are the workers at these industrial sites who are exposed to these chemicals. Each year, 400 to 500 accidents such as suffocation and addiction from exposure to hazardous chemicals due to lack of awareness or careless handling of harmful chemicals occur in domestic workplaces [2]. In addition, the number of occupational diseases occurring from exposure to harmful substances in Korea has nearly doubled from 1959 in 2015 to 4035 in 2019, and various diseases that are difficult to find causes for in the workplace have become a social problem [3].

Korea’s Occupational Safety and Health Act was revised in January 2020 [4]. These revisions included risk assessment, through which the employer identifies harmful hazards caused by construction, machinery, equipment, raw materials, gas, steam, dust, workers’ work behaviors, or other tasks. The employer is then required to assess whether the magnitude of risk that could lead to injury or illness is within an acceptable range, and to take measures, as per legal requirements, to prevent risks or health problems to their workers [5]. This implies that the risk-generating employer should control to reduce the risk “So Far as Reasonably Practicable” (SFARP) and that all risks have been reduced SFARP [6].

The increasing importance of conducting a risk assessment of hazardous substances in the efficient prevention and management of occupational diseases is due to the scientific basis of setting standards [7]. Risk assessment can provide comprehensive and quantitative information on the effects of exposure to hazardous substances in the workplace on the human body, thereby contributing to the acquisition of trust between workers, employers, and governments, and providing reasonable regulatory standards to establish engineering and administrative management goals. Since risk assessment is essential in the management of hazardous substances, relevant academic organizations have worked hard to establish international regulations; developing technologies for risk assessment methods and international cooperation are also critical tasks [8]. Recently, the Occupational Safety and Health Research Institute has been pushing for a project involving the conduct of risk assessment by obtaining data on hazardous substances that are often used in industrial sites but do not have toxic test data to serve as a basis for chemical management at the national level [9]. By conducting this risk assessment of hazardous substances, we can identify the various hazards present in the workplace, analyze and assess the risks that may arise, and formulate appropriate management measures [10]. In Korea, a quantitative risk assessment method called the “Guidelines for Hazard Risk Assessment of Chemicals (KOSHA GUIDE W-6-2016)” was previously used as a method to assess the risk of chemicals. As a qualitative risk assessment measure, Korea Occupational Safety and Health Agency (KOSHA) developed a chemical qualitative risk assessment method (CHARM) based on the UK’s control banding [4,11,12].

The UK developed control banding for practical management recommendations to help small and medium-sized enterprises (SMEs) with their risk assessment of substances and risk management decisions [13]. The qualitative risk assessment method (CHARM) is easier to perform than the existing quantitative risk assessment method, so it is not only easy for an enterprise to follow, but can also be evaluated without direct measurements. It would be more efficient to use qualitative risk assessment methods if the results of this qualitative risk assessment are comparable to those of quantitative risk assessment methods and if similar results are obtained. Therefore, we tried to compare the results obtained after employing qualitative and quantitative risk assessment methods to 36 kinds of hazardous substances requiring management.

## 2. Materials and Methods

### 2.1. Targets of Evaluation

The targets for this study were 36 kinds of hazardous substances requiring management that could help obtain measurement results of the working environment from the KOSHA, as of 2011; information on the 20 substances to be presented as examples is shown in Table 1. Korea is required to measure the working environment approximately twice a year and report the results to the KOSHA. In this study, data on the work environment measurement results for some of the management target substances were used.

It is suggested that the classification labeling of CMR substances is classified according to the standards for classification and labeling of chemical substances and the safety data sheet based on the rules for European Regulation on the Classification, Labeling, and Packaging of substances and mixtures (EU CLP). Accordingly, they are classified into 1A (known to have CMR potential for human is largely based on human evidence), 1B (presumed to have CMR potential for human is largely based on animal evidence), and 2 (suspected human CMR) [4].

### 2.2. Qualitative Risk Assessment 

Qualitative risk assessment followed the CHARM procedure developed by the KOSHA, and its contents are as follows.

If there is a measurement of the working environment for the target substance, the risk is calculated according to the workplace environment result or exposure standards like so:

Risk = Exposure level (Probability) × Hazard level (Severity)(1)

The method of determining the level of exposure in CHARM involves (1) determining whether people had occupational diseases (D1) due to the chemical; when there is an occurrence of occupational diseases, the highest level of exposure level is applied. If there is no occurrence of occupational disease; (2) the work environment measurement results are checked or (3) the handling amount/volatility is checked, and the exposure is classified into one of four levels.

The method for determining the hazard level (severity) involves the following: (1) for CMR substances, the hazard is classified as Grade 4; (2) classifying the severity according to the occupational exposure limit (OEL) based on the Ministry of Employment and Labor in Korea, and (3) classifying the four levels according to the R phrase or H phrase of the Material Safety Data Sheet (MSDS) if a substance does not have OEL. At this time, the probability and the severity are applied in the order of priority Method (1) > Method (2) > Method (3). Risk determination is calculated in 16 grades of risk, as shown in Table 2, according to the exposure and hazard levels, and the level of risk is divided into four levels [14].

### 2.3. Quantitative Risk Assessment 

Quantitative risk assessment involved a four-step risk assessment system for hazard identification, dose-response assessment, exposure assessment, and risk characterization provided by the KOSHA GUIDE W-6-2016. In the dose-response assessment step, appropriate dose-response relationship data based on epidemiological studies and experimental animal data collected in the hazard identification step were used. In the exposure assessment step, the exposure data for each target substance from the 2011 Work Environment Measurement Result, provided by the KOSHA, were used. The central tendency estimate (CTE) and reasonable maximum estimate (RME) for the average daily dose (ADD) and lifetime average daily dose (LADD) for each target substance were calculated using the Monte Carlo Simulation (Crystal ball 11.1, USA). Unit risk (UR) was calculated for 17 carcinogenic substances for which no threshold exist [7,15]. 

For non-carcinogenic substances, if a threshold exists, the reference dose (RfD) or reference concentration (RfC) was calculated using the no observed adverse effect level (NOAEL) or the lowest observed adverse effect level (LOAEL) as in Equation (2). Based on these values, the workplace reference concentration (RfC_work_) was calculated. The hazard quotient (HQ) was calculated by comparing the workplace exposure level values [8].

(2) $$\mathrm{Human}\;\mathrm{dose}\;(\mathrm{RfC}\;\mathrm{or}\;\mathrm{RfD})\;=\;\frac{\mathrm{Experimental}\;\mathrm{dose}\;(\mathrm{NOAEL}\;\mathrm{or}\;\mathrm{LOAEL})}{\mathrm{UF}\;\times \;\mathrm{MF}}$$

where UF is the uncertainty factor, and MF is the modifying factor.

### 2.4. Comparison of Qualitative and Quantitative Assessment

According to the 2019 Guidelines for Risk Assessment developed by the Ministry of Employment and Labor and the Korea Safety and Health Agency, the level of risk can be subdivided into four levels—very high, high, moderate, and low—through a qualitative risk assessment. According to the KOSHA GUIDE W-6-2016, the risk of carcinogenic or non-carcinogenic substances is determined based on 10^−4^ and 1, respectively, through quantitative risk assessment [14,15].

In the case of quantitative risk assessments, it is necessary to classify the risk level by subdividing, in a manner like that used in qualitative risk assessment, from a management perspective.

In this study, to compare the results of the qualitative and quantitative assessments, the results of the quantitative evaluation were subdivided into four levels of risk, as in the qualitative assessments, where the grades of risk are shown in Table 2 as well as the classification of the substances included in each stage.

The risk level was classified by the risk value in the qualitative risk assessment, but the quantitative risk value has not yet been classified by level. However, in this study, the level of quantitative risk assessment was classified to compare it with the qualitative risk assessment value. 

Potential health effects from exposure to chemicals are usually expressed as HQ or HI. HQ is defined as the ratio of the exposure level of the chemical to the reference concentration as follows:

(3) $$\mathrm{Hazard}\;\mathrm{Quotient},\;\mathrm{HQ}\;=\;\frac{E}{\mathrm{RfC}}$$

where E is the chemical exposure level (mg/m^3^) and RfC is the reference concentration (mg/m^3^).

In the case of the HQ, 1 is the management standard; thus, >1 is classified as very high, 0.5 to 1 as high, 0.1 to 0.5 as moderate, and <0.1 as low. In the case of the Excess Cancer Risk (ECR), the MOEL used 10^−4^ as the management criterion; thus, the value was divided by 10^−4^ and evaluated as per the HQ standard.

Table 3 shows that according to the risk assessment guidelines, the risk can be estimated and classified into four levels: low, moderate, high, and very high [12].

## 3. Results

### 3.1. Qualitative Risk Assessment

The 2011 results of the work environment measurement for the target substances are shown in Table 4. When the RME was divided by the OELs, most of them were found to be less than 10%, except for vanadium pentoxide. Since most substances are CMR substances, they are sometimes used to minimize exposure as much as possible at the workplace site, and many of the results of work environment measurements have been measured at less than 10%. If the measurement results are less than 10% of the OEL, exposure assessment by CHARM is low at Class 1.

In Table 5, the hazardous substances requiring management are categorized according to the qualitative risk assessment. In the case of the hazard class, 36 substances were CMR materials, thus, they were classified as Class 4. In the case of the exposure class, most of the results of the work environment measurements were less than 10%, thus, they were classified as Class 1. Vanadium pentoxide, having 12.5%, was classified as Class 2. Therefore, the results of the calculation of the risk considering the hazard and exposure level showed that the qualitative risk assessment value was Grade 4 for 35 substances, except for vanadium pentoxide, which was classified as Grade 8.

### 3.2. Quantitative Risk Assessment

Table 6 shows the results of the quantitative risk assessment of chemical substances requiring management for CTE and RME. As shown in Table 5, there were 17~18 at the low level, eight at the moderate level, four at the high level, and 6~7 at the very high level. The quantitative risk assessment showed that there were seven substances exceeding the HQ values of 1 at the RME level: 1,2,3-Trichloropurophane, ethylene dichloride, 2-methoxyethylacetate, perchloroethylene, nitromethane, pyridine, and vanadium pentoxide. At the CTE level, it was found that six substances, except pyridine, were included. This means that the magnitude of the risk is very high. At the RME level, four substances—acrylonitrile, 1,2-epoxypropane, dichloromethane, cobalt, and their inorganic compounds—had HQ values of 0.5 to 1.0. At the CTE level, four substances were found: acrylonitrile, dichloromethane, pyridine, cobalt, and their inorganic compounds. This can be indicated by the magnitude of the risk being less than 1, but still high. At the RME and CTE levels, the HQ values of 0.1 to 0.5 were shown as diethanolamine, 1,4-dioxane, ethyl acrylate, chlorobenzene, 1,2,2-tetrachloroethane, tetrahydrofuran, 1,1,2-trichloroethylobutone, and eight methyl ketones. These levels were classified as having medium levels of hazard. The HQ values of less than 0.1 were acrylamide, hydrazine, n-hexane, dimethyl aniline, methylene bisphenyl isocyanate, 2-butoxyethanol, methyl bromide, vinyl acetate, cyclohexanone, acetaldehyde, toluene-2,4-diisocyanate, toluene-2,6-diisocyanate, methyl n-butylketone, aniline and homologues, hydrogen peroxide, mercury and its compounds, and titanium dioxide at the RME level. At the CTE level, it was found to be 18 including 1,4-dioxane. It can be said that this level of risk was as low as 10% of the HQ standard 1.

### 3.3. Comparison of Qualitative and Quantitative Assessments

**Figure 1:**
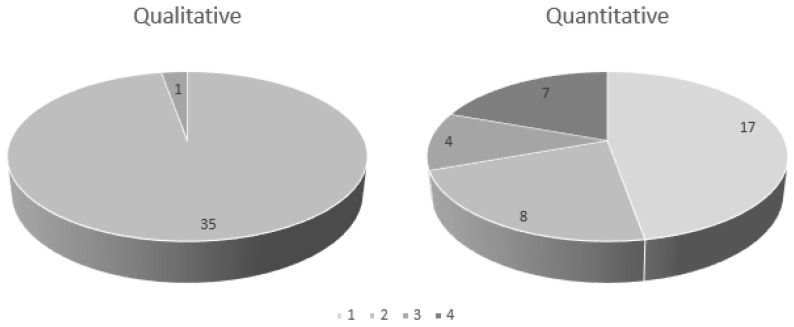
Comparison of the results of the qualitative and quantitative risk assessment of the managed substances.

As shown in Figure 1, according to the results of the qualitative risk assessment, there were 35 substances with moderate risk and one substance with high risk. The results of the quantitative risk assessment revealed that there were eight substances with moderate risk and four substances with high risk. In addition, there were 17 substances with low risk and seven substances with very high risk. As shown in Figure 1, the qualitative risk assessment was found to be relatively evenly distributed compared to the qualitative risk assessment, which showed that most of the work environment measurement results were less than 10%, indicating that it was the first grade. However, because all the substances subject to management were CMR, the hazard level was high, at Grade 5. Eventually, most of the substances were rated as Class 4 for hazard and Class 1 for the possibility of exposure, resulting in a Grade 4 risk. Eventually, it was revealed to be much lower than the highest Grade 16.

On the other hand, the quantitative risk assessment was obtained by dividing the exposure level as per the result of the work environment measurement by the RfD (reference dose) value. Although most work environment measurement results have low values, if the RfD value has a relatively lower value, the HQ value may exceed 1. Therefore, even if the work environment measurement result has a value less than 10% of the OEL, the HQ value may exceed 1 when the RfD value is very low.

## 4. Discussion

The qualitative risk assessment was based on the Control of Substances Hazardous to Health (COSHH) essentials control banding strategy for Health and Safety Executive (HSE) in the UK. The HSE has developed these programs to help SMEs conduct risk assessment for chemical exposure [16]. In Korea, CHARM was developed based on the British COSHH essential control banding [15]. Control banding, developed in the UK, offers a simple method to manage workers’ exposure to chemicals even without workshop exposure data. Control banding for chemical management provides a user-friendly method for workplace management without reliable toxicological and quantitative exposure information [17]. Paik et al. applied the control banding method to assess and manage the risk level of exposure to nanoparticles [18]. Qualitative risk assessment was performed based on the hazards and handling amounts of chemicals. Yang et al. developed an initial risk assessment database from the GHS database based on the type, degree, and handling amounts of chemical hazards, weighted it, and proposed 12 kinds of priority candidates that required hazard and risk assessment [9].

Since all the substances in this study were CMR, there were no low-risk substances because they were all considered Grade 4 in the qualitative risk assessment. In addition, none of the substances had a very high level of risk because there were no substances with a 50% or higher ratio of work environment measurements to OEL. Vanadium pentoxide, with an exposure level of Class 2 in 12.4%, was calculated as Grade 8, indicating that it was high. Excluding vanadium pentoxide, 35 types of target substances were medium risk of Grade 4, as they were ranked Class 1 in the exposure class. In the case of quantitative risk assessment, the RME of the HQ for seven kinds of substances—1,2,3-trichloropurophane, ethylene dichloride, 2-methoxyethylacetate, perchloroethylene, nitromethane, pyridine, and vanadium pentoxide—exceeded 1, which is likely to have harmful effects on the handling worker. Of the 36 target substances, four were at high risk, eight were at moderate risk, and 17 were at low risk. According to the KOSHA’s CHARM, if the risk level is high or moderate, work should continue, but risk reduction activities should be carried out when there is no current risk. While the qualitative assessments indicated that all 36 types of target substances were high or medium, the quantitative assessment results identified only about 12 substances in these categories (33%). Therefore, we conclude that the quantitative risk level range setting presented in this study lacks feasibility and is less relevant to qualitative and quantitative risk assessments. It will be difficult to predict quantitative risk assessment results through qualitative risk assessments or vice versa.

In addition, since all of the substances included in this study were CMR substances, risk was calculated as Grade 4 in the qualitative evaluation. Therefore, further investigation on non-CMR substances, which are likely to have relatively few hazards, is warranted.

The limitation of this study is that the 36 kinds of target substances were CMR because of the limited range of workplace environment measurement data obtained from KOSHA. Among the chemical substances requiring management, many non-CMR substances exist. Therefore, there is a need to expand and evaluate materials other than CMR. It was found that CHARM, a qualitative risk assessment method, differed from quantitative risk assessment. There is a need for research to narrow this gap and improve the method so that the results are similar to the quantitative risk assessment results.

## 5. Conclusions

Risk assessment of hazardous substances is increasingly important to efficiently prevent and manage various diseases or accidents caused by increased amounts of hazardous substances in the workplace. In this study, we conducted a risk assessment of 36 kinds of hazardous substances requiring management using both qualitative and quantitative assessment methods. The qualitative risk assessment was performed by multiplying the exposure level class and the hazard class according to the CHARM of the KOSHA. The quantitative risk assessment involved a 4-step risk assessment system presented in the KOSHA GUIDE W-6-2016. In quantitative assessments, we presented a new method of classifying risk levels into four steps, much like qualitative assessments.

Vanadium pentoxide was ranked as Class 2 in the exposure class with a Grade 8 risk, indicating that it was high. Excluding vanadium pentoxide, 35 types of target substances had a moderate risk of Grade 4 as they were ranked as Class 1 in the exposure class. In the case of quantitative risk assessment, seven kinds of substances showed very high-risk levels, four kinds showed high levels, eight kinds showed moderate levels, and 17 kinds showed low risk levels in the workplace exposure level of RME. According to the CHARM, it is stipulated that if the risk level is high or moderate, work should continue, but risk reduction activities should be carried out when there is no current risk. As a result of the qualitative evaluation, all 36 kinds of target substances showed high or moderate risk, but among the quantitative evaluation results, the risk of 12 substances, which was about 33% of the target substances, fell into these categories.

Therefore, the results of this study show that the relationship between the results of the qualitative and quantitative risk assessment of chemical substances requiring management is not high. It is still difficult to predict the result of quantitative risk assessment through qualitative risk assessment or vice versa. After a qualitative risk assessment, a quantitative risk assessment is necessary for a high tier assessment. In addition, there is need for research on how the relationship between assessments in two ways can be enhanced.

## Figures and Tables

**Table 1:** Hazards of chemical substances requiring management in a workplace environment.

No.	Substance Name	CAS No.	CMR * Information			Skin Effect
			Carcinogenicity	Mutagenicity	Reproductive Toxicity	
1	Acrylamide	79-06-1	1B	1B	2	Skin
2	1,2,3-Trichloropropane	96-18-4	1B		1B	Skin
3	Ethylene dichloride	107-06-2	1B			
4	Acrylonitrile	107-13-1	1B			Skin
5	2-Methoxyethyl acetate	110-49-6			1B	Skin
6	1,2-Epoxypropane	75-56-9	1B	1B		
7	Perchloroethylene	127-18-4	1B			
8	Hydrazine	302-01-2	1B			Skin
9	n-Hexane	110-54-3			2	
10	Nitromethane	75-52-5	2			
11	Dimethylaniline(N,N-Dimethylaniline)	121-69-7	2			Skin
12	Diethanolamine	111-42-2	2			
13	1,4-Dioxane	123-91-1	2			Skin
14	Dichloromethane	75-09-2	2			
15	Methylene bisphenyl isocyanate	101-68-8	2			
16	2-Butoxyethanol	111-76-2	2			Skin
17	Methyl bromide	74-83-9		2		Skin
18	Vinyl acetate	108-05-4	2			
19	Vanadium pentoxide	1314-62-1	2	2	2	
20	Acetaldehyde	75-07-0	2			

* CMR: Carcinogenicity, Mutagenicity, Reproductive toxicity.

**Table 2:** Classification of risk assessment level.

Grades of Risk		Hazard Quotient	Permissible Status
12 to 16	Very high	>1	Unacceptable
5 to 11	High	0.5 to 1	
3 to 4	Moderate	0.1 to 0.5	Acceptable or Unacceptable (CMR) *
1 to 2	Low	<0.1	Acceptable

* CMR: Carcinogenicity, Mutagenicity, Reproductive toxicity.

**Table 3:** Estimation of the risk level of chemical substances.

	Hazard Level	Very High	High	Moderate	Low
Exposure Level	Level	4	3	2	1
Very high	4	16	12	8	4
High	3	12	9	6	3
Moderate	2	8	6	4	2
Low	1	4	3	2	1

**Table 4:** Workplace environment measurement results of the target substances.

No.	Substance Name	Measured Numbers	Exposure Values (mg/m^3^)		RME/OEL (%)
			CTE *	RME **	
1	Acrylamide	452	1.56 × 10^−4^	1.95 × 10^−4^	0.7
2	1,2,3-Trichloropropane	55	7.41 × 10^−1^	8.64 × 10^−1^	8.6
3	Ethylene dichloride	365	3.82 × 10^−1^	4.55 × 10^−1^	4.6
4	Acrylonitrile	905	3.39 × 10^−2^	4.11 × 10^−2^	2.1
5	2-Methoxyethyl acetate	871	9.10 × 10^−2^	9.97 × 10^−2^	2.0
6	1,2-Epoxypropane	45	5.17 × 10^−2^	6.62 × 10^−2^	3.3
7	Perchloroethylene	2317	5.59 × 10^−1^	7.01 × 10^−1^	2.8
8	Hydrazine	233	2.25 × 10^−4^	2.77 × 10^−4^	0.6
9	n-Hexane	16754	7.66 × 10^−1^	8.35 × 10^−1^	1.7
10	Nitromethane	16	9.93 × 10^−1^	1.28 × 10^0^	6.4
11	Dimethylaniline(N,N-Dimethylaniline)	68	2.27 × 10^−4^	3.36 × 10^−4^	0.0
12	Diethanolamine	893	8.60 × 10^−3^	9.65 × 10^−3^	2.1
13	1,4-Dioxane	765	2.36 × 10^−1^	2.96 × 10^−1^	1.5
14	Dichloromethane	7629	3.01 × 10^0^	3.61 × 10^0^	0.7
15	Methylene bisphenyl isocyanate	2395	1.03 × 10^−4^	1.86 × 10^−4^	3.7
16	2-Butoxyethanol	8673	2.59 × 10^−1^	3.13 × 10^−1^	1.6
17	Methyl bromide	59	1.63 × 10^−5^	2.34 × 10^−5^	0.0
18	Vinyl acetate	2117	3.14 × 10^−1^	3.63 × 10^−1^	3.6
19	Vanadium pentoxide	38	6.17 × 10^−3^	6.86 × 10^−3^	13.7
20	Acetaldehyde	43	2.58 × 10^−2^	3.28 × 10^−2^	1.0

* CTE: central tendency estimates, ** RME: reasonable maximum estimates.

**Table 5:** Qualitative risk assessment results for hazardous substances requiring management.

Grades of Risk		Qualitative Assessment	Permissible Status
		Substances (No.)	
12 to 16	Very high	0	Unacceptable
5 to 11	High	1	
3 to 4	Medium	35	Acceptable or Unacceptable (CMR) *
1 to 2	Low	0	Acceptable

* CMR: Carcinogenicity, Mutagenicity, Reproductive toxicity.

**Table 6:** Results of the quantitative risk assessment of hazardous substances requiring management.

Grades of Risk	Substances (No.)		HQ
	CTE *	RME **	
12~16	6	7	>1 × 10^0^
5~11	4	4	0.5~1
3~4	8	8	0.1~0.5
1~2	18	17	<0.1

* CTE: central tendency estimates, ** RME: reasonable maximum estimates.

## Data Availability

Not applicable.
